# Influence of Frailty on Outcome in Older Patients Undergoing Non-Cardiac Surgery - A Systematic Review and Meta-Analysis

**DOI:** 10.14336/AD.2019.1024

**Published:** 2020-10-01

**Authors:** Elke K.M Tjeertes, Joris M.K van Fessem, Francesco U.S Mattace-Raso, Anton G.M Hoofwijk, Robert Jan Stolker, Sanne E Hoeks

**Affiliations:** ^1^Department of Anesthesiology, Erasmus MC University Medical Center, Rotterdam, the Netherlands; ^2^Department of Internal Medicine, Division of Geriatric Medicine, Erasmus MC University Medical Center, Rotterdam, the Netherlands; ^3^Department of Surgery, Zuyderland Medical Center, Geleen, the Netherlands

**Keywords:** frailty, surgery, outcome, older patients, non-cardiac surgery

## Abstract

Frailty is increasingly recognized as a better predictor of adverse postoperative events than chronological age. The objective of this review was to systematically evaluate the effect of frailty on postoperative morbidity and mortality. Studies were included if patients underwent non-cardiac surgery and if frailty was measured by a validated instrument using physical, cognitive and functional domains. A systematic search was performed using EMBASE, MEDLINE, Web of Science, CENTRAL and PubMed from 1990 - 2017. Methodological quality was assessed using an assessment tool for prognosis studies. Outcomes were 30-day mortality and complications, one-year mortality, postoperative delirium and discharge location. Meta-analyses using random effect models were performed and presented as pooled risk ratios with confidence intervals and prediction intervals. We included 56 studies involving 1.106.653 patients. Eleven frailty assessment tools were used. Frailty increases risk of 30-day mortality (31 studies, 673.387 patients, risk ratio 3.71 [95% CI 2.89-4.77] (PI 1.38-9.97; I2=95%) and 30-day complications (37 studies, 627.991 patients, RR 2.39 [95% CI 2.02-2.83). Risk of 1-year mortality was threefold higher (six studies, 341.769 patients, RR 3.40 [95% CI 2.42-4.77]). Four studies (N=438) reported on postoperative delirium. Meta-analysis showed a significant increased risk (RR 2.13 [95% CI 1.23-3.67). Finally, frail patients had a higher risk of institutionalization (10 studies, RR 2.30 [95% CI 1.81- 2.92]). Frailty is strongly associated with risk of postoperative complications, delirium, institutionalization and mortality. Preoperative assessment of frailty can be used as a tool for patients and doctors to decide who benefits from surgery and who doesn’t.

Life expectancy has increased with the focus on the quality of added life-years [1]. This prolonged life expectancy has created an increased demand for surgical care of the elderly [2, 3].

Several studies have described age as an independent risk factor for postoperative morbidity and mortality in both cardiac and non-cardiac surgery [4-7]. Advantages in operative techniques and perioperative management seem to improve outcome and multiple studies have even demonstrated an improved quality of life and enhancement of functional status after cardiac surgery in octogenarians [8-10]. Despite these improvements in perioperative care, postoperative adverse effects still remain more common in older patients when compared to the younger ones [5, 11]. Adequate risk assessment integrates surgical factors and factors that describe the biological status of the patient, rather than age alone, as age per se seems to be responsible for only a small increase in adverse events [3, 12].

Recently the concept of frailty has come into view [2]. Frailty can be defined as a clinically recognizable state of increased vulnerability resulting from aging-associated lack of physiological reserve and decline in function across multiple physiologic systems [13]. Focus on and optimization of frail patients can contribute to a reduced postoperative morbidity and thereby to better outcome in the older surgical population [2]. Globally, the World Health Organisation has recently developed recommendations on integrated care for older patients in order to maintain their physical and cognitive functions [14].

In order to adequately inform our patients of significant perioperative risks, additional information on frailty as a risk factor influencing postoperative outcome is essential. During the preoperative assessment, this information can guide the clinician in shared decision making on whether the older patient benefits from surgery or not. The aim of this study was to evaluate the predictive role of frailty on postoperative outcomes after non-cardiac surgery by conducting a systematic review and meta-analysis of literature.

## METHODS

### Search Strategy

A search of literature was performed and reported according to the Preferred Reporting Items for Systematic Reviews and Meta-analyses (PRISMA) statement and MOOSE criteria [15]. The objective was to find all studies on frail patients undergoing non-cardiac surgery, correlating their age and its subsequent risk factors to postoperative morbidity and mortality. The systematic Internet based search was performed using EMBASE, MEDLINE, Web of Science, Cochrane Central Register of Controlled Trials (CENTRAL) and PubMed. Full electronic searches can be found in Supplementary Table. 1. In addition, we screened the reference section of all articles included in this review. The search was limited to original articles, human subjects and articles published from January 1990 - December 2017.

### Publication selection

Two reviewers independently (EKMT and JMKvF) screened potentially relevant articles from the initial search, first by title and abstract and later on by full text. Any disagreements between the two reviewers were resolved by discussion and consensus with a third reviewer (SH). Studies were found eligible for inclusion if their subjects underwent non-cardiac surgery and if frailty was measured by a frailty instrument using at least physical, cognitive and functional domains. Also, the relationship between frailty and primary outcomes of 30-day mortality, or 30-day complications should be evaluated, with stratification of the outcome (frail versus non-frail). Studies were excluded if they were review articles, case reports, editorials or comments, or if full text was not available. Duplicate articles were removed during the initial search.

### Data Extraction

The following data were gathered from eligible publications: publication date, study design, sample size, type of surgery, proportion of females, mean age, the frailty score and outcome. Outcome was measured by the following adverse events: 30-day mortality, 30-day complications, one-year mortality, manifestation of postoperative delirium (POD) and discharge to a specialized facility. 30-day complications are generally defined as suggested by the Clavien-Dindo classification system[16]; otherwise the authors should have predefined this outcome. Postoperative delirium was defined as a temporary state of confusion and diagnosis made with validated delirium screening tools or by a geriatric expert team [17]. Discharge destination was defined as “home”, or “not able to return home”. Furthermore, surgical procedures were categorised according to the ESC/ESA Guidelines [18] and divided into low-, intermediate- and high-risk procedures. Occasionally, the surgical risk category was documented as “mixed surgical population”. A subanalysis per surgery type was performed to better understand the effect of frailty according to the surgical risk category. Where absolute data were not presented in table or text and authors could not be reached, when possible, data were extracted from figures using WebPlotDigitizer (version, 2.6.8).

### Assessment of quality and possible biases

Two reviewers performed assessment of quality. In case of disagreement a third reviewer was consulted. The quality assessment tool for prognosis studies as proposed by Hayden et al. was used for the appraisal of all included studies [19]. This tool focuses on six areas of potential bias; first *study participation* (i.e. the study sample represents the population of interest on key characteristics), second *study attrition* (i.e. whether the study was able to obtain a complete follow up), third *prognostic factor measurement* (i.e. a clear definition or description of the prognostic factor measured is provided), fourth *outcome measurement*(i.e. a clear definition of the outcome of interest), fifth *confounding measurement and account* (i.e. important potential confounders are appropriately accounted for) and sixth *analysis*(i.e. the statistical analysis is appropriate for the design of the study). After the evaluation of these six areas of potential bias, all studies were subsequently divided According to the Quality in Prognosis Study Tool into good (11 or 12 points), fair (9 or 10 points) and poor (< 9 points) quality.

### Statistical methods

Numerical values reported by the studies were used for analysis. In some cases, further calculation was required for ascertaining outcomes. In the studies using the modified frailty index (mFI) patients were categorized into two groups: “not frail” (mFI < 0.27), or “frail” (mFI ≥ 0.27). The decision to divide patients into those categories was based on thresholds most commonly used to indicate the presence of frailty and was made before analysis. In the remaining studies, using ten different frailty instruments, outcome was also dichotomized according to predefined criteria as “not frail” or “frail”. Random effects models for meta-analysis were used because of the large expected heterogeneity in determinant and other study characteristics. The primary outcome measures 30-day mortality and 30-day complications were stratified by frailty score. Furthermore, a subanalysis per surgery type was performed to better understand the effect of frailty according to the surgical risk category. Effect estimates are presented as pooled risk ratios (RR) with 95% confidence intervals (CI’s). Robust meta-analytic conclusions of prognosis studies will be more appropriately signaled when prediction intervals are provided [20]. Thus, to further account for between-study heterogeneity, 95% prediction interval (PI) were also estimated, which evaluates the uncertainty of the effect that would be expected in a new study addressing the same association [21]. I^2^ statistic was calculated, which is the percentage of variation across studies due to heterogeneity rather than random error. Since all reported outcomes were adverse events, a positive relative risk indicates that frailty is associated with worse patient outcome. A meta-regression analysis was carried out to assess the influence of the patient’s mean age (using mean or median age of the study populations as a proxy) on 30-day mortality. Finally, an additional sensitivity analysis was performed (excluding studies using ACS-NSQIP database) to circumvent the issue of possible duplicate cases and demonstrate the effect of frailty on postoperative outcome.

Data gathering and data analysis was performed using Excel (version 14.7.2) and Rstudio (version 1.1.463) respectively.

**Figure 1.**
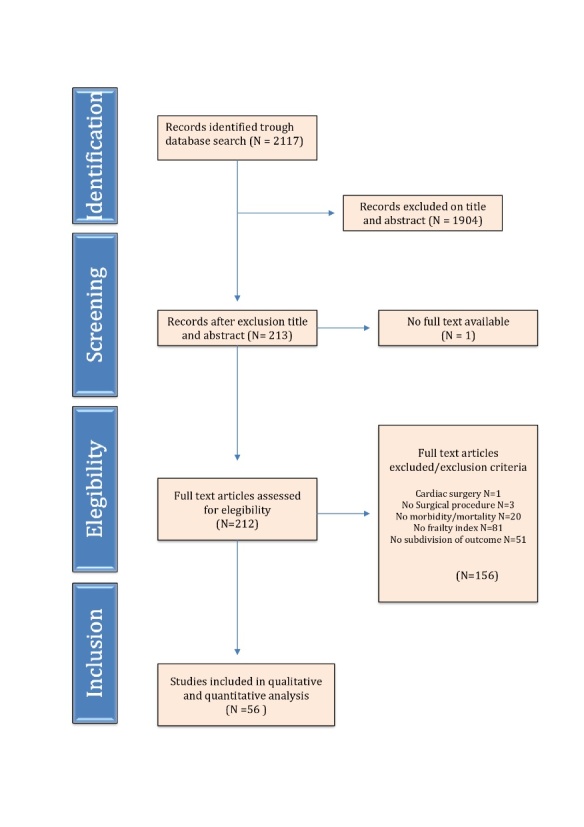
PRISMA flowchart for study selection. This flowchart depicts the flow of information trough different phases of the systematic research.

## RESULTS

Initial literature search identified 2117 manuscripts as potentially relevant. Of these, 1904 were excluded due to unrelated research questions or study type. Full text was not available in one study; therefore 212 full text articles were thoroughly screened for eligibility. A total of 56 studies were found suitable for this systematic review. Figure 1 shows the search strategy flow chart.

### Frailty assessment tools

A total of eleven different frailty assessment tools were used. The majority of studies (twenty-four) used the Modified Frailty Index (mFI), created by Saxton and Velanovich [22]. The mFI consists of eleven variables present in the Canadian Study on Health and Aging Frailty Index, as well as in the American College of Surgeons National Surgical Quality Improvement Program (ACS NSQIP) dataset [23, 24]. Variations on the Fried Frailty Criteria [25] were used in eleven studies, where frailty was defined by identifying unintentional weight loss, exhaustion, low energy expenditure, low grip strength and slow walking speed. Frailty assessment tools were often based on comprehensive geriatric assessments, which can be derived from questionnaires or patient files, including the Frailty Index and the Groningen Frailty Indicator. Supplementary Fig. 2 provides a detailed description of all frailty assessment tools used in this review.

**Figure 2.**
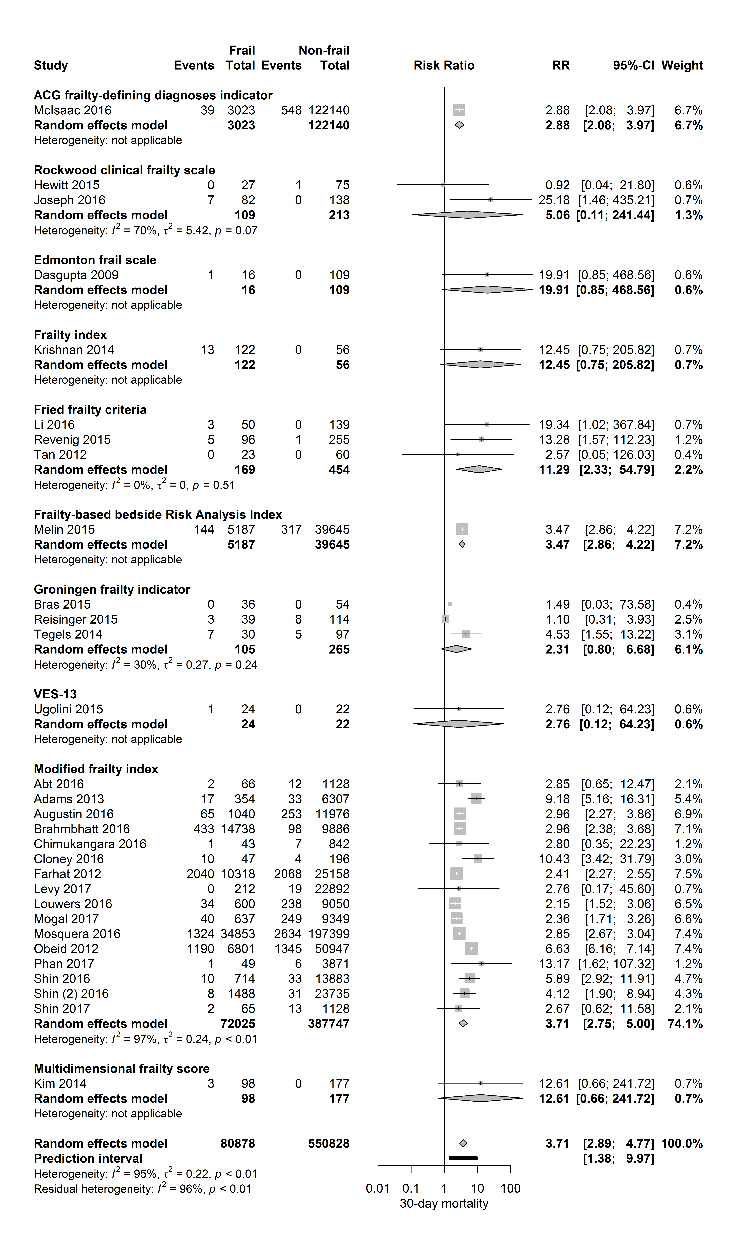
Forest plot 30-day mortality per frailty score. The number of events (deaths) and the total number of patients are shown for both frail and non-frail patients, stratified per frailty assessment tool.

**Figure 3.**
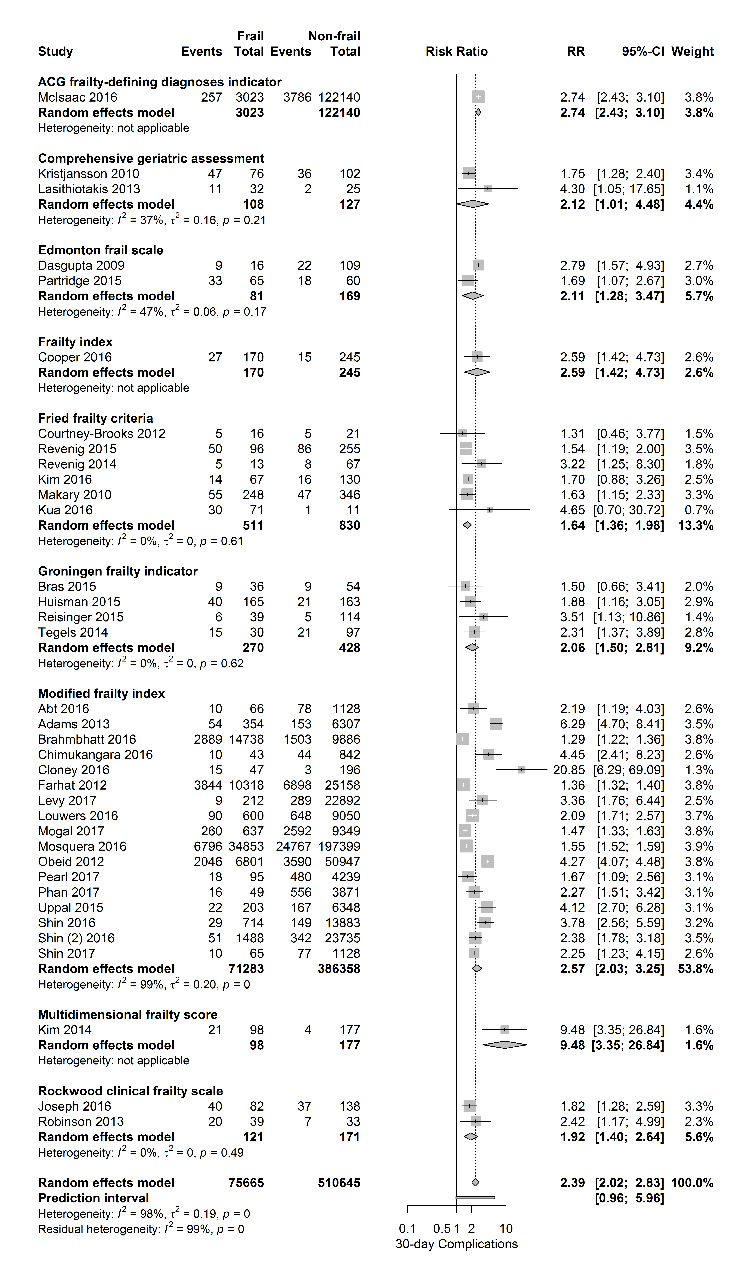
Forest plot postoperative complications per frailty score. The number of events (complications) and the total number of patients are shown for both frail and non-frail patients, stratified per frailty assessment tool.

### Quality assessment

The quality assessment of the included studies is provided in Supplementary Fig. 3 and table 1 provides a summary of our appraisal. Study participation was adequately described in 37 studies. The study attrition - referring to the response rate and attempts to collect information on patients who were lost to follow up - was adequately defined in 40 studies. Prognostic factors were clearly defined or described in most studies (86%). Ninety-one percent of studies provided a clear definition of the outcome of interest. When summarizing, 95% of all studies included were of at least fair quality, with more than half assessed as good quality.

**Table 1:** Study demographics and method of determining frailty.

Author	N	Setting	Period	Design	Type of surgery	Frailty score	Definition of complication	Quality
Abt	1193	Multicenter cohort study (NSQIP)	2006-2013	Prospective	Head and neck cancer surgery	Modified frailty index	CD 4	Good
Adams	6727	Multicenter cohort study (NSQIP)	2005-2010	Prospective	Head and neck cancer surgery	Modified frailty index	CD 4 or 5	Good
Arya	23027	Multicenter cohort study (NSQIP)	2005-2012	Prospective	Vascular surgery (Open or EVAR)	Modified frailty index	CD 4	Good
Augustin	13020	Multicenter cohort study (NSQIP)	2005-2010	Prospective	Pancreatic resections	Modified frailty index	CD 4	Good
Brahmbhatt	24645	Multicenter cohort study (NSQIP)	2005-2012	Prospective	Infrainguinal vascular surgery	Modified frailty index	CD 4	Good
Bras	90	Single-center cohort study	2008-2013	Retrospective	Surgery for head and neck cancer	Groningen frailty indicator	CD ≥ 2	Fair
Chappidi	2679	Multicenter cohort study (NSQIP)	2011-2013	Prospective	Radical cystectomy	Modified frailty index	CD 4 or 5	Good
Chimukangara	885	Multicenter cohort study (NSQIP)	2011-2013	Prospective	Paraesofageal hernia repair	Modified frailty index	CD ≥ 3	Fair
Cloney	243	Multicenter cohort study (NSQIP)	2000-2012	Prospective	Glioblastoma surgery	Modified frailty index	Complications (Glioma Outcomes Project System)	Fair
Cooper	415	Multicenter cohort study	2010-2013	Prospective	General and orthopedic surgery	Frailty phenotype; frailty index	Major complications	Fair
Courtney-Brooks	37	Single-center cohort study	2011	Prospective	Surgery for gynecologic cancer	Fried frailty criteria	Surgical complications (NSQIP)	Fair
Dale	76	Single-center cohort study	2007-2011	Prospective	Pancreaticoduodenectomy	4 (of 5) components of Fried frailty criteria; VES-13	CD ≥ 3	Fair
Dasgupta	125	Single-center cohort study	2002-2003	Prospective	Elective noncardiac surgery (82%) orthopedic)	Edmonton frail scale	Cardiac - / pulmonary comlications, POD	Fair
Farhat	35334	Multicenter cohort study (NSQIP)	2005-2009	Prospective	Emergency general surgery	Modified frailty index	Any complication (not mortality)	Fair
Flexman	52671	Multicenter cohort study (NSQIP)	2006-2012	Prospective	Spine surgery	Modified frailty index	Major complications	Good
Hewitt	102	Multicenter cohort study	2013	Prospective	Emergency general surgery	Rockwood clinical frailty scale	Not reported	Fair
Huisman	328	Multicenter cohort study	2008-2012	Prospective	Surgery for solid tumors	Groningen frailty indicator; VES-13	CD ≥ 3	Good
Joseph	220	Single-center cohort study	2012-2014	Prospective	Emergency general surgery	Rockwood clinical frailty scale	Surgical complications (NSQIP)	Fair
Kenig	184	Single-center cohort study	2013-2014	Prospective	Emergency abdominal surgery	VES-13, GFI; Rockwood; Balducci; TRST; Geriatric-8	Any complication (CD)	Fair
Kim	197	Single-center cohort study	2012-2014	Prospective	Elective noncardiac surgery	Fried frailty criteria	Surgical complications (NSQIP)	Good
Kim	275	Single-center cohort study	2011-2012	Prospective	Elective intermediate-risk or high-risk surgery	Multidimensional frailty score	Surgical complications (NSQIP)	Good
Krishnan	178	Single-center cohort study	2011	Prospective	Low trauma hip fracture surgery	Frailty index	Not reported	Poor
Kristjansson	178	Multicenter cohort study	2008-2011	Prospective	Elective surgery for colorectal cancer	Comprehensive geriatric assessment	CD ≥ 2	Good
Kua	82	Single-center cohort study	2013	Prospective	Hip fracture surgery	Edmonton frail scale; (modified) Fried frailty criteria	Any complication	Fair
Lascano	41681	Multicenter cohort study (NSQIP)	2005-2013	Prospective	Surgery for urologic cancer	Modified frailty index	CD 4	Good
Lasithiotakis	57	Single-center cohort study	2008-2011	Prospective	Elective laparoscopic cholecystectomy	Comprehensive geriatric assessment	Any complication	Poor
Leung	63	Single-center cohort study	2007	Prospective	Noncardiac surgery	Fried frailty criteria	Not reported	Fair
Levy	23104	Multicenter cohort study (NSQIP)	2008 to 2014	Prospective	Robot-assisted radical prostatectomy	Modified frailty index	CD 4	Good
Li	189	Single-center cohort study	Not reported	Prospective	Major intra-abdominal surgery	Fried frailty criteria	CD	Fair
Louwers	10300	Multicenter cohort study (NSQIP)	2005-2011	Prospective	Hepatectomy	Modified frailty index	CD 4	Good
Makary	594	Single-center cohort study	2005-2006	Prospective	Elective surgery	Fried frailty criteria	Surgical complications (NSQIP)	Good
McAdams-DeMarco	537	Single-center cohort study	2008-2013	Prospective	Kidney transplant surgery	Fried frailty criteria	Not reported	Fair
McIsaac	202811	Single-center cohort study	2002-2012	Retrospective	Major elective noncardiac surgery	ACG frailty-defining diagnoses indicator	Not reported	Good
McIsaac	125163	Single-center cohort study	2003-2012	Retrospective	Total joint arthroplasty	ACG frailty-defining diagnoses indicator	ICU-admission	Good
Melin	44832	Multicenter cohort study (NSQIP)	2005-2011	Prospective	Carotid endarterectomy	Frailty-based bedside Risk Analysis Index	Not reported	Fair
Mogal	9986	Multicenter cohort study (NSQIP)	2005-2012	Prospective	Pancreaticoduodenectomy	Modified frailty index	CD 3 or 4	Good
Mosquera	232352	Multicenter cohort study (NSQIP)	2005-2012	Prospective	elective high-risk surgery	Modified frailty index	Major and minor complications	Fair
Neuman	12979	Single-center cohort study	1992-2005	Retrospective	Elective colorectal cancer surgery	ACG frailty-defining diagnoses indicator	Readmission within 30 days	Fair
Obeid	58448	Multicenter cohort study (NSQIP)	2005-2009	Prospective	Laparoscopic and open colectomy	Modified frailty index	CD 4 or 5	Fair
Partridge	125	Single-center cohort study	2011	Prospective	Arterial vascular surgery	Edmonton frail scale	Composite postoperative complications	Fair
Pearl	4330	Multicenter cohort study (NSQIP)	2011-2014	Prospective	Radical cystectomy	Modified frailty index	Major in-hospital complications	Good
Phan	3920	Multicenter cohort study (NSQIP)	2010-2014	Prospective	Elective anterior lumbar interbody fusion (ALIF) surgery	Modified frailty index	Any complication	Good
Reisinger	159	Single-center cohort study	2010-2012	Prospective	Colorectal surgery	Groningen frailty indicator	Sepsis	Good
Revenig	351	Single-center cohort study	Not reported	Prospective	Major intra-abdominal surgery	Fried frailty criteria	CD 1-4	Fair
Revenig	80	Single-center cohort study	Not reported	Prospective	Intra-abdominal minimally invasive surgery	Fried frailty criteria	CD 1-4	Fair
Revenig	189	Single-center cohort study	Not reported	Prospective	Major intra-abdominal surgery	Fried frailty criteria	Any complication	Good
Robinson	72	Single-center cohort study	2007-2010	Prospective	Colorectal surgery	Rockwood clinical frailty scale	Any postoperative complication (VASQIP)	Fair
Shin	6148 ACDF; 817 PCF	Multicenter cohort study (NSQIP)	2005-2012	Prospective	Cervical spine fusion; anterior cervical discectomy and fusion or posterior cervical fusion	Modified frailty index	CD 4	Good
Shin	14583 THA; 25223 TKA	Multicenter cohort study (NSQIP)	2005-2012	Prospective	Total hip and knee arthroplasty	Modified frailty index	CD 4	Good
Suskind	95108	Multicenter cohort study (NSQIP)	2007-2013	Prospective	Common urological surgery	Modified frailty index	Major and minor complications	Good
Suskind	20794	Multicenter cohort study (NSQIP)	2011-2013	Prospective	Inpatient urological surgery	Modified frailty index	Not reported	Good
Tan	83	Multicenter cohort study	2008-2010	Prospective	Colorectal surgery	Fried frailty criteria	CD ≥ 2	Fair
Tegels	127	Single-center cohort study	2005-2012	Retrospective	Surgery for gastric cancer	Groningen frailty indicator	CD ≥ 3	Fair
Tsiouris	1940	Multicenter cohort study (NSQIP)	2005-2010	Prospective	Open lobectomy	Modified frailty index	CD 4	Good
Ugolini	46	Single-center cohort study	2009-2012	Prospective	Elective colorectal cancer surgery	Groningen frailty indicator; VES-13	Not reported	Poor
Uppal	6551	Multicenter cohort study (NSQIP)	2008-2011	Prospective	Surgery for gynecologic cancer	Modified frailty index	CD 4 and 5	Good

Abbreviations: CD = Cavien-Dindo classification of surgical complications; NSQIP = National Surgical Quality Improvement Program

### Postoperative outcome predicted by frailty

Table 1 shows the details of study demographics and methods of frailty measurement. In the selected studies, fifty-one were of prospective design and sample size ranged from 37 - 232 352 patients. Gender distribution was reported in 93% of the studies with a proportion of females ranging from 0% in the study of Levy et al, describing a male population undergoing robot assisted radical prostatectomies, until 100% in the study of Courtney-Brooks et al, describing complications in elderly women undergoing gynecologic oncology surgery. Twenty-seven studies investigated the effect of frailty in oncological surgery (predominantly abdominal cancer surgery), four studies in vascular surgery, nine in orthopedic surgery, eleven in elective general surgery (predominantly intermediate - and high-risk surgery), four in emergency surgery and one study in transplant surgery. Thirty-one studies investigated the influence of frailty on 30-day mortality. Figure 2 shows a forest plot of this primary outcome with a pooled RR of 3.71 [95% CI 2.89-4.77] (PI 1.38-9.97; I2=95%) for frail patients compared to those who were not frail. The 95% prediction interval also showed exclusion of the null value.

**Figure 4.**
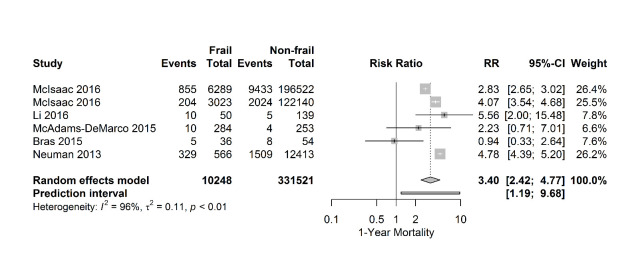
Forest plot 1-year mortality. The number of events (one-year mortality) and the total number of patients are depicted for frail and non-frail patients.

Stratified for frailty assessment tool, the association of frailty and 30-day mortality was observed according to the ACG frailty-defining diagnosis indicator, Fried frailty criteria, Frailty-based Risk Analysis Index and the Modified Frailty Index.

Figure 3 shows the relationship between frailty and the occurrence of postoperative complications, stratified for frailty assessment tool. This adverse outcome was evaluated in 37 papers. Table 1 shows the predefined 30-day complications reported by the authors, in most cases defined as suggested by the Clavien-Dindo classification system. Overall, a positive relationship between frailty and 30-day complications with a pooled RR of 2.39 [95% CI 2.02-3.07] was observed (PI 0.96-5.69; I2=98%), regardless of the frailty score used.

Stratified per surgical risk category, pooled RR’s for 30-day mortality were 2.75 [95% CI 2.48-3.05] for high-risk surgery (4 studies), RR 4.79 [95% CI 3.42-6.70] for intermediate-risk surgery (18 studies) and RR 3.06 [95% CI 2.35-3.97] for mixed surgical population (8 studies). The association of frailty and the primary outcome 30-day complications was also stratified per surgical risk category and again a positive relationship was observed with pooled RR’s of 1.62 [95% CI 1.43 -1.82] for high-risk surgery (3 studies) and RR 2.94 [95% CI 2.44-3.54] for intermediate-risk surgery (24 studies).

Six studies investigated the association between frailty and one-year mortality (Fig. 4). In most of these studies, frailty increases the risk of one-year mortality with a pooled consequent risk ratio of 3.40 [95% CI 2.42-4.77], (PI 1.19- 9.68; I2=96%).

**Figure 5.**
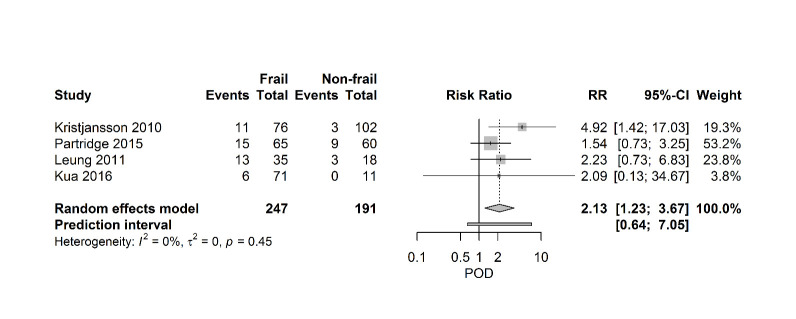
Forest plot postoperative delirium. The number of events (delirium) and the total number of patients are depicted for frail and non-frail patients.

Figure 5 shows a forest plot, which summarizes the relationship between frailty and postoperative delirium. Four studies (438 patients) describe a positive relationship between frailty and POD with a pooled RR of 2.13 [95% CI 1.23-3.67], (PI 0.64- 7.05; I2=0%).

**Figure 6.**
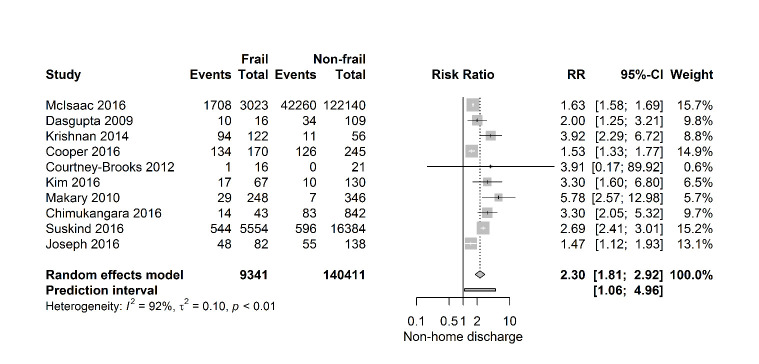
Forest plot discharge to specialized facility. The number of events (discharge to a specialized facility) and the total number of patients are depicted for frail and non-frail patients.

Figure 6 shows that frail patients seem to struggle to return to their own home, as these patients, described in ten studies (149 752 patients), have a twofold higher risk of being discharged to a specialized facility after surgery (RR 2.30 [95% CI 1.81-2.92]), (PI 1.06- 4.96; I2=92%). Just like in 30-day mortality and one-year mortality, the 95% prediction interval for postoperative discharge location showed exclusion of the null value.

A meta-regression analysis investigating showed no influence of age on primary outcome. Finally, to circumvent the issue of possible duplicate cases, the additional sensitivity analysis excluding studies using ACS-NSQIP database, showed an overall pooled RR of 3.62 [CI 95% 2.21-5.92] (PI 1.46-8.98; I2=14%) for 30-day mortality

## DISCUSSION

Since life expectancy keeps rising, the number of frail patients being offered for surgical treatment will dramatically increase. Frail patients are vulnerable and may excessively decompensate after stressors such as surgery, because of their lack of physiological reserve [13].

In this systematic review and meta-analysis, we found frailty to be a strong predictor of post surgical complications, delirium, institutionalization and all-cause mortality. After reviewing fifty-six articles, 30-day mortality shows the strongest association with preoperative frailty with almost 4 times increased risk.

Our results are congruent with several other reviews investigating the effect of frailty on postoperative outcome. [26-30] However, most of the previous studies focused on specific age groups, specific types of surgery, or specific frailty assessment tool. Therefore, extrapolations to a heterogeneous group of elderly and multimorbid patients should be limited.

The strength of the present study is the extensiveness of the search, the inclusion of different validated frailty scores and the inclusion of different types of non-cardiac surgery, both elective and acute. The quality of this meta-analysis is dependent on the quality of the studies reviewed. Of all studies included 95% were of at least fair quality, with more than half assessed as good quality. Ninety-one percent of all studies were prospectively designed.

Recently, relevant developments have been made towards methodological frameworks, in order to improve the reliability and applicability of prediction studies [31]. Although the authors found improved reporting standards in the last decade, poor reporting and poor methods are still a topic of concern and likely to limit the reliability in this type of clinical research.

The studies in this review and meta-analysis describe eleven different frailty assessment tools. Moreover, the surgical procedures included could basically be divided into six different groups, which will have contributed to the heterogeneity. Heterogeneity, as assessed with I2, t2, Cochran’s Q and prediction intervals, was estimated as a high degree of statistical heterogeneity. Importantly, the association between frailty and outcome seems robust throughout the reviewed articles regardless of the frailty assessment tool used. Furthermore, prediction intervals of 30-day mortality, one-year mortality and postoperative discharge location showed exclusion of the null value, which strengthens our findings.

A plausible explanation may be the fact that frailty was consistently measured by instruments using physical, cognitive and functional domains. Studies using only measurements of body composition or patients’ phenotype, such as sarcopenia, hypoalbuminemia or cachexia were not included, as these studies did not use an established frailty assessment tool. The frailty instrument used in most studies was the modified frailty index (mFI), which has been validated as a reliable assessment tool in several studies [32-36]. It should be recommended that future studies focus on using a standardized, robust and validated frailty assessment tool, which is time-efficient and suitable for the medical staff to be conducted at patient’s bedside.

Limitations of this study are those commonly seen with systematic reviews and meta-analysis. Hence, the results of this review and meta-analysis should be interpreted with caution. Besides the heterogeneity, another possible limitation is a variation among studies in the definition of discharge location. Despite these small differences, ten studies confirm that frail patients, when compared to healthier counterparts, struggle to return to their own home. Unfortunately, in many countries, availability of beds and nursing staff in specialized facilities are a topic of current concern. To overcome this limitation the need for rehabilitation or nursing home placement was defined as “not able to return home”. Comparable heterogeneity was found within the definition of postoperative complications. Although most authors defined 30-day complications as suggested by the Clavien-Dindo classification system, others used the American College of Surgery National Surgical Quality Improvement Program definition, or other standardized complication definitions. It should be recommended that future studies in the area of frailty use a standardized postoperative complication definition as this might create a more accurate comparison. The International Consortium for Health Outcomes Measurement (ICHOM) recently developed the first global standard set of outcome measures in older persons. Their effort towards standardization of outcome measures can possibly improve care pathways and quality of care [37].

Although we have performed an exhaustive literature search, the broad scope of our research question could have resulted in the omission of some studies.

Many studies in this systematic review and meta-analysis are observational registry studies, but several studies have derived their outcomes from clinical trials. Since many studies have used the ACS NSQIP database, there may be studies, which are double counted from the same cohort of patients. However, table 1 shows that most of these studies observed different subgroups of patients, as well as different timeframes and kinds of surgical specialisms. Additionally, the sensitivity analysis we have performed, excluding studies using ACS-NSQIP database, demonstrated a positive relationship between frailty and primary outcomes. Finally, subgroup analyses gave insight in the heterogeneity among the types of surgery and different frailty assessment tools, but this stratification has the drawback of small groups.

In a previous study we have found that the occurrence of postoperative complications is an important prognostic factor of late mortality [38]. Efforts to improve postoperative outcome have predominantly focused on enhanced recovery protocols and the improvement of surgical and anesthetic techniques [39, 40]. The concept of prehabilitation is a modern and proactive approach, based on the principle that structured exercise over a period of weeks leads to a better cardiovascular, respiratory and muscular condition. Optimization of patients’ functional capacity may provide a physiological buffer and enables the patient to better withstand the stress of surgery [39, 41, 42].

Preoperative identification of frail patients provides an opportunity for prehabilitation, which subsequently may lead to reduced postoperative morbidity. Besides prehabilitation, regionalization in health care might improve surgical outcome in complex oncological surgery. Regionalization is about enabling appropriate allocation and integration of health resources, focusing on the local populations needs. Frail patients may benefit from high-volume hospitals with high-volume surgeons in so called centers of excellence [43].

This study demonstrates that the presence of preoperative frailty increases the risk of adverse outcome after non-cardiac surgery. It should be noted that heterogeneity of the frailty scores is high, but associations with postoperative outcome are robust. Frailty status should be considered to be part of the preoperative screening, at least in patients who seem to have a lack of physiological reserve. Identification of potentially reversible health deficits is important, as may provide an opportunity to optimize patients’ clinical condition prior to surgery. Conversely, irreversible frailty should be taken most seriously, as it can guide both clinician and patient in their decision making on whether the patient benefits from surgery or not.

## Supplementary Materials

The Supplemenantry data can be found online at: www.aginganddisease.org/EN/10.14336/AD.2019.1024.

## References

[1] Mangano DT (2004). Perioperative medicine: NHLBI working group deliberations and recommendations. J Cardiothorac Vasc Anesth, 18:1-6. 14973791 10.1053/j.jvca.2003.10.002

[2] Partridge JS, Harari D, Dhesi JK (2012). Frailty in the older surgical patient: a review. Age Ageing, 41:142-147. 22345294 10.1093/ageing/afr182

[3] Kristensen SD, Knuuti J, Saraste A, Anker S, Botker HE, De Hert S, et al (2014). 2014 ESC/ESA Guidelines on non-cardiac surgery: cardiovascular assessment and management: The Joint Task Force on non-cardiac surgery: cardiovascular assessment and management of the European Society of Cardiology (ESC) and the European Society of Anaesthesiology (ESA). Eur J Anaesthesiol, 31:517-573. 25127426 10.1097/EJA.0000000000000150

[4] Lee DH, Buth KJ, Martin BJ, Yip AM, Hirsch GM (2010). Frail patients are at increased risk for mortality and prolonged institutional care after cardiac surgery. Circulation, 121:973-978. 20159833 10.1161/CIRCULATIONAHA.108.841437

[5] Polanczyk CA, Marcantonio E, Goldman L, Rohde LE, Orav J, Mangione CM, et al (2001). Impact of age on perioperative complications and length of stay in patients undergoing noncardiac surgery. Ann Intern Med, 134:637-643. 11304103 10.7326/0003-4819-134-8-200104170-00008

[6] Browner WS, Li J, Mangano DT (1992). In-hospital and long-term mortality in male veterans following noncardiac surgery. The Study of Perioperative Ischemia Research Group. JAMA, 268:228-232. 1608142

[7] Arozullah AM, Khuri SF, Henderson WG, Daley J, Participants in the National Veterans Affairs Surgical Quality Improvement P (2001). Development and validation of a multifactorial risk index for predicting postoperative pneumonia after major noncardiac surgery. Ann Intern Med, 135:847-857. 11712875 10.7326/0003-4819-135-10-200111200-00005

[8] Huber CH, Goeber V, Berdat P, Carrel T, Eckstein F (2007). Benefits of cardiac surgery in octogenarians--a postoperative quality of life assessment. Eur J Cardiothorac Surg, 31:1099-1105. 17369044 10.1016/j.ejcts.2007.01.055

[9] Fruitman DS, MacDougall CE, Ross DB (1999). Cardiac surgery in octogenarians: can elderly patients benefit? Quality of life after cardiac surgery. Ann Thorac Surg, 68:2129-2135. 10616989 10.1016/s0003-4975(99)00818-8

[10] Filsoufi F, Rahmanian PB, Castillo JG, Chikwe J, Silvay G, Adams DH (2007). Results and predictors of early and late outcomes of coronary artery bypass graft surgery in octogenarians. J Cardiothorac Vasc Anesth, 21:784-792. 18068053 10.1053/j.jvca.2007.08.007

[11] Baskett R, Buth K, Ghali W, Norris C, Maas T, Maitland A, et al (2005). Outcomes in octogenarians undergoing coronary artery bypass grafting. CMAJ, 172:1183-1186. 15851711 10.1503/cmaj.1041342 PMC557069

[12] Sundermann S, Dademasch A, Rastan A, Praetorius J, Rodriguez H, Walther T, et al (2011). One-year follow-up of patients undergoing elective cardiac surgery assessed with the Comprehensive Assessment of Frailty test and its simplified form. Interact Cardiovasc Thorac Surg, 13:119-123; discussion 123. 21378017 10.1510/icvts.2010.251884

[13] Xue QL (2011). The frailty syndrome: definition and natural history. Clin Geriatr Med, 27:1-15. 21093718 10.1016/j.cger.2010.08.009 PMC3028599

[14] 2017 In Integrated Care for Older People: Guidelines on Community-Level Interventions to Manage Declines in Intrinsic Capacity. Geneva. 29608259

[15] Liberati A, Altman DG, Tetzlaff J, Mulrow C, Gotzsche PC, Ioannidis JP, et al (2009). The PRISMA statement for reporting systematic reviews and meta-analyses of studies that evaluate healthcare interventions: explanation and elaboration. BMJ, 339:b2700. 19622552 10.1136/bmj.b2700 PMC2714672

[16] Dindo D, Demartines N, Clavien PA (2004). Classification of surgical complications: a new proposal with evaluation in a cohort of 6336 patients and results of a survey. Ann Surg, 240:205-213. 15273542 10.1097/01.sla.0000133083.54934.ae PMC1360123

[17] De J, Wand AP (2015). Delirium Screening: A Systematic Review of Delirium Screening Tools in Hospitalized Patients. Gerontologist, 55:1079-1099. 26543179 10.1093/geront/gnv100

[18] Kristensen SD, Knuuti J (2014). New ESC/ESA Guidelines on non-cardiac surgery: cardiovascular assessment and management. Eur Heart J, 35:2344-2345. 25104785 10.1093/eurheartj/ehu285

[19] Hayden JA, Cote P, Bombardier C (2006). Evaluation of the quality of prognosis studies in systematic reviews. Ann Intern Med, 144:427-437. 16549855 10.7326/0003-4819-144-6-200603210-00010

[20] Graham PL, Moran JL (2012). Robust meta-analytic conclusions mandate the provision of prediction intervals in meta-analysis summaries. J Clin Epidemiol, 65:503-510. 22265586 10.1016/j.jclinepi.2011.09.012

[21] IntHout J, Ioannidis JP, Rovers MM, Goeman JJ (2016). Plea for routinely presenting prediction intervals in meta-analysis. BMJ Open, 6:e010247. 10.1136/bmjopen-2015-010247 PMC4947751 27406637

[22] Velanovich V, Antoine H, Swartz A, Peters D, Rubinfeld I (2013). Accumulating deficits model of frailty and postoperative mortality and morbidity: Its application to a national database. J Surg Res, 183:104-110. 23415494 10.1016/j.jss.2013.01.021

[23] Searle SD, Mitnitski A, Gahbauer EA, Gill TM, Rockwood K (2008). A standard procedure for creating a frailty index. BMC Geriatr, 8:24. 18826625 10.1186/1471-2318-8-24 PMC2573877

[24] Fuchshuber PR, Greif W, Tidwell CR, Klemm MS, Frydel C, Wali A, et al (2012). The power of the National Surgical Quality Improvement Program--achieving a zero pneumonia rate in general surgery patients. Perm J, 16:39-45. 22529758 10.7812/tpp/11-127 PMC3327110

[25] Fried LP, Tangen CM, Walston J, Newman AB, Hirsch C, Gottdiener J, et al (2001). Frailty in older adults: evidence for a phenotype. J Gerontol A Biol Sci Med Sci, 56:M146-156. 11253156 10.1093/gerona/56.3.m146

[26] Hewitt J, Long S, Carter B, Bach S, McCarthy K, Clegg A (2018). The prevalence of frailty and its association with clinical outcomes in general surgery: a systematic review and meta-analysis. Age Ageing, 47:793-800. 30084863 10.1093/ageing/afy110

[27] Oakland K, Nadler R, Cresswell L, Jackson D, Coughlin PA (2016). Systematic review and meta-analysis of the association between frailty and outcome in surgical patients. Ann R Coll Surg Engl, 98:80-85. 26741674 10.1308/rcsann.2016.0048 PMC5210486

[28] Lin HS, Watts JN, Peel NM, Hubbard RE (2016). Frailty and post-operative outcomes in older surgical patients: a systematic review. BMC Geriatr, 16:157. 27580947 10.1186/s12877-016-0329-8 PMC5007853

[29] Panayi AC, Orkaby AR, Sakthivel D, Endo Y, Varon D, Roh D, et al (2018). Impact of frailty on outcomes in surgical patients: A systematic review and meta-analysis. Am J Surg. 10.1016/j.amjsurg.2018.11.020 PMC6536365 30509455

[30] Wang J, Zou Y, Zhao J, Schneider DB, Yang Y, Ma Y, et al (2018). The Impact of Frailty on Outcomes of Elderly Patients After Major Vascular Surgery: A Systematic Review and Meta-analysis. Eur J Vasc Endovasc Surg, 56:591-602. 30122332 10.1016/j.ejvs.2018.07.012

[31] Bouwmeester W, Zuithoff NP, Mallett S, Geerlings MI, Vergouwe Y, Steyerberg EW, et al (2012). Reporting and methods in clinical prediction research: a systematic review. PLoS Med, 9:1-12. 10.1371/journal.pmed.1001221 PMC3358324 22629234

[32] Ehlert BA, Najafian A, Orion KC, Malas MB, Black JH, Abularrage CJ (2016). Validation of a modified Frailty Index to predict mortality in vascular surgery patients. J Vasc Surg, 63:1595e1592-1601e1592. 26926932 10.1016/j.jvs.2015.12.023

[33] Abt NB, Richmon JD, Koch WM, Eisele DW, Agrawal N (2016). Assessment of the predictive value of the modified frailty index for Clavien-Dindo grade IV critical care complications in major head and neck cancer operations. JAMA Otolaryngol Head Neck Surg, 142:658-664. 27258927 10.1001/jamaoto.2016.0707

[34] Ali R, Schwalb JM, Nerenz DR, Antoine HJ, Rubinfeld I (2016). Use of the modified frailty index to predict 30-day morbidity and mortality from spine surgery. J Neurosurg Spine, 25:537-541. 27153143 10.3171/2015.10.SPINE14582

[35] Tsiouris A, Hammoud ZT, Velanovich V, Hodari A, Borgi J, Rubinfeld I (2013). A modified frailty index to assess morbidity and mortality after lobectomy. J Surg Res, 183:40-46. 23273884 10.1016/j.jss.2012.11.059

[36] Uppal S, Igwe E, Rice LW, Spencer RJ, Rose SL (2015). Frailty index predicts severe complications in gynecologic oncology patients. Gynecol Oncol, 137:98-101. 25602715 10.1016/j.ygyno.2015.01.532

[37] Akpan A, Roberts C, Bandeen-Roche K, Batty B, Bausewein C, Bell D, et al (2018). Standard set of health outcome measures for older persons. BMC Geriatr, 18:36. 29394887 10.1186/s12877-017-0701-3 PMC5797357

[38] Tjeertes EK, Ultee KH, Stolker RJ, Verhagen HJ, Bastos Goncalves FM, Hoofwijk AG, et al (2016). Perioperative Complications are Associated With Adverse Long-Term Prognosis and Affect the Cause of Death After General Surgery. World J Surg, 40:2581-2590. 27302465 10.1007/s00268-016-3600-4 PMC5073115

[39] Wynter-Blyth V, Moorthy K (2017). Prehabilitation: preparing patients for surgery. BMJ, 358:j3702. 28790033 10.1136/bmj.j3702

[40] Adamina M, Kehlet H, Tomlinson GA, Senagore AJ, Delaney CP (2011). Enhanced recovery pathways optimize health outcomes and resource utilization: a meta-analysis of randomized controlled trials in colorectal surgery. Surgery, 149:830-840. 21236454 10.1016/j.surg.2010.11.003

[41] Moorthy K, Wynter-Blyth V (2017). Prehabilitation in perioperative care. Br J Surg, 104:802-803. 28300279 10.1002/bjs.10516

[42] Carli F, Scheede-Bergdahl C (2015). Prehabilitation to enhance perioperative care. Anesthesiol Clin, 33:17-33. 25701926 10.1016/j.anclin.2014.11.002

[43] Lumpkin S, Stitzenberg K (2018). Regionalization and Its Alternatives. Surg Oncol Clin N Am, 27:685-704. 30213413 10.1016/j.soc.2018.05.009

[44] Krishnan M, Beck S, Havelock W, Eeles E, Hubbard RE, Johansen A (2014). Predicting outcome after hip fracture: Using a frailty index to integrate comprehensive geriatric assessment results. Age Ageing, 43:122-126. 23832264 10.1093/ageing/aft084

[45] Cooper Z, Rogers SO, Ngo L, Guess J, Schmitt E, Jones RN, et al (2016). Comparison of Frailty Measures as Predictors of Outcomes After Orthopedic Surgery. J Am Geriatr Soc, 64:2464-2471. 27801939 10.1111/jgs.14387 PMC5173406

[46] Slaets JP (2006). Vulnerability in the elderly: frailty. Med Clin North Am, 90:593-601. 16843764 10.1016/j.mcna.2006.05.008

[47] Bras L, Peters TTA, Wedman J, Plaat BEC, Witjes MJH, van Leeuwen BL, et al (2015). Predictive value of the Groningen Frailty Indicator for treatment outcomes in elderly patients after head and neck, or skin cancer surgery in a retrospective cohort. Clin Otolaryngol, 40:474-482. 25754107 10.1111/coa.12409

[48] Huisman MG, Audisio RA, Ugolini G, Montroni I, Vigano A, Spiliotis J, et al (2015). Screening for predictors of adverse outcome in onco-geriatric surgical patients: A multicenter prospective cohort study. Eur J Surg Oncol, 41:844-851. 25935371 10.1016/j.ejso.2015.02.018

[49] Reisinger KW, Van Vugt JLA, Tegels JJW, Snijders C, Hulsewé KWE, Hoofwijk AGM, et al (2015). Functional compromise reflected by sarcopenia, frailty, and nutritional depletion predicts adverse postoperative outcome after colorectal cancer surgery. Ann Surg, 261:345-352. 24651133 10.1097/SLA.0000000000000628

[50] Tegels JJW, de Maat MFG, Hulsewé KWE, Hoofwijk AGM, Stoot JHMB (2014). Value of Geriatric Frailty and Nutritional Status Assessment in Predicting Postoperative Mortality in Gastric Cancer Surgery. J Gastrointest Surg, 18:439-446. 24420730 10.1007/s11605-013-2443-7

[51] Ugolini G, Pasini F, Ghignone F, Zattoni D, Reggiani MLB, Parlanti D, et al (2015). How to select elderly colorectal cancer patients for surgery: a pilot study in an Italian academic medical center. Cancer Biol Med, 12:302-307. 26779367 10.7497/j.issn.2095-3941.2015.0084 PMC4706530

[52] Kenig J, Zychiewicz B, Olszewska U, Barczynski M, Nowak W (2015). Six screening instruments for frailty in older patients qualified for emergency abdominal surgery. Arch Gerontol Geriatr, 61:437-442. 26211706 10.1016/j.archger.2015.06.018

[53] Kua J, Ramason R, Rajamoney G, Chong MS (2016). Which frailty measure is a good predictor of early post-operative complications in elderly hip fracture patients? Arch Orthop Trauma Surg, 136:639-647. 26980097 10.1007/s00402-016-2435-7

[54] Li JL, Henderson MA, Revenig LM, Sweeney JF, Kooby DA, Maithel SK, et al (2016). Frailty and one-year mortality in major intra-abdominal operations This study was presented at the World Congress of Endourology in London in October 2015. J Surg Res, 203:507.e501-512.e501. 27087115 10.1016/j.jss.2016.03.007

[55] Revenig LM, Canter DJ, Kim S, Liu Y, Sweeney JF, Sarmiento JM, et al (2015). Report of a simplified frailty score predictive of short-term postoperative morbidity and mortality. J Am Coll Surg, 220:904-911.e901. 25907870 10.1016/j.jamcollsurg.2015.01.053

[56] Revenig LM, Canter DJ, Master VA, Maithel SK, Kooby DA, Pattaras JG, et al (2014). A prospective study examining the association between preoperative frailty and postoperative complications in patients undergoing minimally invasive surgery. J Endourol, 28:476-480. 24308497 10.1089/end.2013.0496

[57] Tan KY, Kawamura YJ, Tokomitsu A, Tang T (2012). Assessment for frailty is useful for predicting morbidity in elderly patients undergoing colorectal cancer resection whose comorbidities are already optimized. Am J Surg, 204:139-143. 22178483 10.1016/j.amjsurg.2011.08.012

[58] Kim S, Marsh AP, Rustowicz L, Roach C, Leng XI, Kritchevsky SB, et al (2016). Self-reported mobility in older patients predicts early postoperative outcomes after elective noncardiac surgery. Anesthesiology, 124:815-825. 26978144 10.1097/ALN.0000000000001011 PMC5201172

[59] Leung JM, Tsai TL, Sands LP (2011). Preoperative frailty in older surgical patients is associated with early postoperative delirium. Anesth Analg, 112:1199-1201. 21372278 10.1213/ANE.0b013e31820c7c06 PMC3081949

[60] Makary MA, Segev DL, Pronovost PJ, Syin D, Bandeen-Roche K, Patel P, et al (2010). Frailty as a Predictor of Surgical Outcomes in Older Patients. J. Am. Coll. Surg., 210:901-908. 10.1016/j.jamcollsurg.2010.01.028 20510798

[61] McAdams-Demarco MA, Law A, King E, Orandi B, Salter M, Gupta N, et al (2015). Frailty and mortality in kidney transplant recipients. Am J Transplant, 15:149-154. 25359393 10.1111/ajt.12992 PMC4332809

[62] Revenig LM, Canter DJ, Taylor MD, Tai C, Sweeney JF, Sarmiento JM, et al (2013). Too frail for surgery? Initial results of a large multidisciplinary prospective study examining preoperative variables predictive of poor surgical outcomes. J Am Coll Surg, 217:665-670.e661. 24054409 10.1016/j.jamcollsurg.2013.06.012

[63] Courtney-Brooks M, Tellawi AR, Scalici J, Duska LR, Jazaeri AA, Modesitt SC, et al (2012). Frailty: An outcome predictor for elderly gynecologic oncology patients. Gynecol Oncol, 126:20-24. 22522190 10.1016/j.ygyno.2012.04.019

[64] Adams P, Ghanem T, Stachler R, Hall F, Velanovich V, Rubinfeld I (2013). Frailty as a predictor of morbidity and mortality in inpatient head and neck surgery. JAMA Otolaryngol Head Neck Surg, 139:783-789. 23949353 10.1001/jamaoto.2013.3969

[65] Arya S, Kim SI, Duwayri Y, Brewster LP, Veeraswamy R, Salam A, et al (2015). Frailty increases the risk of 30-day mortality, morbidity, and failure to rescue after elective abdominal aortic aneurysm repair independent of age and comorbidities. J Vasc Surg, 61:324-331. 25312534 10.1016/j.jvs.2014.08.115

[66] Augustin T, Burstein MD, Schneider EB, Morris-Stiff G, Wey J, Chalikonda S, et al (2016). Frailty predicts risk of life-threatening complications and mortality after pancreatic resections. Surgery, 160:987-996. 27545992 10.1016/j.surg.2016.07.010

[67] Brahmbhatt R, Brewster LP, Shafii S, Rajani RR, Veeraswamy R, Salam A, et al (2016). Gender and frailty predict poor outcomes in infrainguinal vascular surgery. J Surg Res, 201:156-165. 26850197 10.1016/j.jss.2015.10.026

[68] Chappidi MR, Kates M, Patel HD, Tosoian JJ, Kaye DR, Sopko NA, et al (2016). Frailty as a marker of adverse outcomes in patients with bladder cancer undergoing radical cystectomy. Urol Oncol Semin Orig Invest. 10.1016/j.urolonc.2015.12.010 PMC4875870 26899289

[69] Chimukangara M, Frelich MJ, Bosler ME, Rein LE, Szabo A, Gould JC (2016). The impact of frailty on outcomes of paraesophageal hernia repair. J Surg Res, 202:259-266. 27229099 10.1016/j.jss.2016.02.042 PMC4884326

[70] Cloney M, D'Amico R, Lebovic J, Nazarian M, Zacharia BE, Sisti MB, et al (2016). Frailty in Geriatric Glioblastoma Patients: A Predictor of Operative Morbidity and Outcome. World Neurosurg, 89:362-367. 26775233 10.1016/j.wneu.2015.12.096

[71] Farhat JS, Velanovich V, Falvo AJ, Horst HM, Swartz A, Patton JH Jr, et al (2012). Are the frail destined to fail? Frailty index as predictor of surgical morbidity and mortality in the elderly. J Trauma Acute Care Surg, 72:1526-1531. 22695416 10.1097/TA.0b013e3182542fab

[72] Flexman AM, Charest-Morin R, Stobart L, Street J, Ryerson CJ (2016). Frailty and postoperative outcomes in patients undergoing surgery for degenerative spine disease. Spine J, 16:1315-1323. 27374110 10.1016/j.spinee.2016.06.017

[73] Lascano D, Pak JS, Kates M, Finkelstein JB, Silva M, Hagen E, et al (2015). Validation of a frailty index in patients undergoing curative surgery for urologic malignancy and comparison with other risk stratification tools. Urol Oncol Semin Orig Invest, 33:426.e421-426.e412. 10.1016/j.urolonc.2015.06.002 PMC4584178 26163940

[74] Levy I, Finkelstein M, Bilal KH, Palese M (2017). Modified frailty index associated with Clavien-Dindo IV complications in robot-assisted radical prostatectomies: A retrospective study. Urol Oncol Semin Orig Invest. 10.1016/j.urolonc.2017.01.005 28190748

[75] Louwers L, Schnickel G, Rubinfeld I (2016). Use of a simplified frailty index to predict Clavien 4 complications and mortality after hepatectomy: Analysis of the National Surgical Quality Improvement Project database. Am J Surg, 211:1071-1076. 26800866 10.1016/j.amjsurg.2015.09.015

[76] Mogal H, Vermilion SA, Dodson R, Hsu FC, Howerton R, Shen P, et al (2017). Modified Frailty Index Predicts Morbidity and Mortality After Pancreaticoduodenectomy. Ann Surg Oncol:1-8. 10.1245/s10434-016-5715-0 PMC7064816 28058551

[77] Mosquera C, Spaniolas K, Fitzgerald TL (2016). Impact of frailty on surgical outcomes: The right patient for the right procedure. Surgery, 160:272-280. 27267548 10.1016/j.surg.2016.04.030

[78] Obeid NM, Azuh O, Reddy S, Webb S, Reickert C, Velanovich V, et al (2012). Predictors of critical care-related complications in colectomy patients using the National Surgical Quality Improvement Program: Exploring frailty and aggressive laparoscopic approaches. J Trauma Acute Care Surg, 72:878-883. 22491599 10.1097/TA.0b013e31824d0f70

[79] Pearl JA, Patil D, Filson CP, Arya S, Alemozaffar M, Master VA, et al (2017). Patient Frailty and Discharge Disposition Following Radical Cystectomy. Clin Genitourin Cancer. 10.1016/j.clgc.2016.12.013 28139446

[80] Phan K, Kim JS, Lee NJ, Somani S, Di Capua J, Kothari P, et al (2017). Frailty is associated with morbidity in adults undergoing elective anterior lumbar interbody fusion (ALIF) surgery. Spine J, 17:538-544. 27989724 10.1016/j.spinee.2016.10.023

[81] Suskind AM, Jin C, Cooperberg MR, Finlayson E, Boscardin WJ, Sen S, et al (2016). Preoperative Frailty Is Associated With Discharge to Skilled or Assisted Living Facilities After Urologic Procedures of Varying Complexity. Urology, 97:25-32. 27392651 10.1016/j.urology.2016.03.073 PMC5477056

[82] Suskind AM, Walter LC, Jin C, Boscardin J, Sen S, Cooperberg MR, et al (2016). Impact of frailty on complications in patients undergoing common urological procedures: A study from the American College of Surgeons National Surgical Quality Improvement database. BJU Int. 10.1111/bju.13399 PMC4833543 26691588

[83] Shin JI, Keswani A, Lovy AJ, Moucha CS (2016). Simplified Frailty Index as a Predictor of Adverse Outcomes in Total Hip and Knee Arthroplasty. J Arthroplasty, 31:2389-2394. 27240960 10.1016/j.arth.2016.04.020

[84] Shin JI, Kothari P, Phan K, Kim JS, Leven D, Lee NJ, et al (2017). Frailty Index as a Predictor of Adverse Postoperative Outcomes in Patients Undergoing Cervical Spinal Fusion. Spine, 42:304-310. 27379416 10.1097/BRS.0000000000001755

[85] Rolfson DB, Majumdar SR, Tsuyuki RT, Tahir A, Rockwood K (2006). Validity and reliability of the Edmonton Frail Scale. Age Ageing, 35:526-529. 16757522 10.1093/ageing/afl041 PMC5955195

[86] Dasgupta M, Rolfson DB, Stolee P, Borrie MJ, Speechley M (2009). Frailty is associated with postoperative complications in older adults with medical problems. Arch Gerontol Geriatr, 48:78-83. 18068828 10.1016/j.archger.2007.10.007

[87] Partridge JSL, Fuller M, Harari D, Taylor PR, Martin FC, Dhesi JK (2015). Frailty and poor functional status are common in arterial vascular surgical patients and affect postoperative outcomes. Int J Surg, 18:57-63. 25907322 10.1016/j.ijsu.2015.04.037

[88] Rockwood K, Song X, MacKnight C, Bergman H, Hogan DB, McDowell I, et al (2005). A global clinical measure of fitness and frailty in elderly people. CMAJ, 173:489-495. 16129869 10.1503/cmaj.050051 PMC1188185

[89] Hewitt J, Moug SJ, Middleton M, Chakrabarti M, Stechman MJ, McCarthy K (2015). Prevalence of frailty and its association with mortality in general surgery. Am J Surg, 209:254-259. 25173599 10.1016/j.amjsurg.2014.05.022

[90] Joseph B, Zangbar B, Pandit V, Fain M, Mohler MJ, Kulvatunyou N, et al (2016). Emergency General Surgery in the Elderly: Too Old or Too Frail? Presented orally at the Surgical Forum of the American College of Surgeons 100th Annual Clinical Congress, San Francisco, CA, October 2014. J Am Coll Surg, 222:805-813. 27113515 10.1016/j.jamcollsurg.2016.01.063

[91] Robinson TN, Wu DS, Pointer L, Dunn CL, Cleveland JC Jr, Moss M (2013). Simple frailty score predicts postoperative complications across surgical specialties. Am J Surg, 206:544-550. 23880071 10.1016/j.amjsurg.2013.03.012 PMC3788864

[92] Saliba D, Elliott M, Rubenstein LZ, Solomon DH, Young RT, Kamberg CJ, et al (2001). The Vulnerable Elders Survey: a tool for identifying vulnerable older people in the community. J Am Geriatr Soc, 49:1691-1699. 11844005 10.1046/j.1532-5415.2001.49281.x

[93] Dale W, Hemmerich J, Kamm A, Posner MC, Matthews JB, Rothman R, et al (2014). Geriatric assessment improves prediction of surgical outcomes in older adults undergoing pancreaticoduodenectomy: A prospective cohort study. Ann Surg, 259:960-965. 24096757 10.1097/SLA.0000000000000226 PMC10157800

[94] Lieberman R AC, Weiner JP 2003 Development and Evaluation of the Johns Hopkins University Risk Adjustment Models for Medicare ? Choice Plan Payment.: Baltimore, MD; Johns Hopkins University.

[95] Neuman HB, Weiss JM, Leverson G, O’Connor ES, Greenblatt DY, Loconte NK, et al (2013). Predictors of Short-Term Postoperative Survival after Elective Colectomy in Colon Cancer Patients ≥80 Years of Age. Ann Surg Oncol, 20:1427-1435. 23292483 10.1245/s10434-012-2721-8 PMC3799845

[96] McIsaac DI, Bryson GL, van Walraven C (2016). Association of Frailty and 1-Year Postoperative Mortality Following Major Elective Noncardiac Surgery: A Population-Based Cohort Study. JAMA Surg. 10.1001/jamasurg.2015.5085 26791334

[97] McIsaac DI, Beaule PE, Bryson GL, Van Walraven C (2016). The impact of frailty on outcomes and healthcare resource usage after total joint arthroplasty: a population-based cohort study. Bone Joint J, 98-B:799-805. 27235523 10.1302/0301-620X.98B6.37124

[98] Makary MA, Segev DL, Pronovost PJ, Syin D, Bandeen-Roche K, Patel P, et al (2010). Frailty as a predictor of surgical outcomes in older patients. J Am Coll Surg, 210:901-908. 20510798 10.1016/j.jamcollsurg.2010.01.028

[99] Melin AA, Schmid KK, Lynch TG, Pipinos II, Kappes S, Longo GM, et al (2015). Preoperative frailty risk analysis index to stratify patients undergoing carotid endarterectomy. J Vasc Surg, 61:683-689. 25499711 10.1016/j.jvs.2014.10.009

[100] Balducci L, Beghe C (2000). The application of the principles of geriatrics to the management of the older person with cancer. Crit Rev Oncol Hematol, 35:147-154. 10960797 10.1016/s1040-8428(00)00089-5

[101] Kristjansson SR, Nesbakken A, Jordhøy MS, Skovlund E, Audisio RA, Johannessen HO, et al (2010). Comprehensive geriatric assessment can predict complications in elderly patients after elective surgery for colorectal cancer: A prospective observational cohort study. Crit Rev Oncol Hematol, 76:208-217. 20005123 10.1016/j.critrevonc.2009.11.002

[102] Lasithiotakis K, Petrakis J, Venianaki M, Georgiades G, Koutsomanolis D, Andreou A, et al (2013). Frailty predicts outcome of elective laparoscopic cholecystectomy in geriatric patients. Surg Endosc Interv Tech, 27:1144-1150. 10.1007/s00464-012-2565-0 23052539

[103] Kim SW, Han HS, Jung HW, Kim KI, Hwang DW, Kang SB, et al (2014). Multidimensional Frailty Score for the Prediction of Postoperative Mortality Risk. JAMA Surg., 149:633-640. 24804971 10.1001/jamasurg.2014.241

