# Supplementary material for: Influence of Frailty on Outcome in Older Patients Undergoing Non-Cardiac Surgery - A Systematic Review and Meta-Analysis
Source: Aging Dis. 2020 Oct 1;11(5):1276–90. doi: 10.14336/AD.2019.1024 (PMC7505262; doi:10.14336/AD.2019.1024)
Supplement: Supplementary file 1 — The Supplemenantry data can be found online at: www.aginganddisease.org/EN/10.14336/AD.2019.1024. [file AD-11-5-1276-suppl.pdf]

SUPPLEMENTARY DATA

Influence of Frailty on Outcome in Older Patients Undergoing Non-Cardiac Surgery – A Systematic Review and Meta-Analysis

Elke K.M. Tjeertes[1], Joris M.K. van Fessem[1], Francesco U.S. Mattace-Raso[2], Anton G.M. Hoofwijk[3], Robert Jan Stolker[1], Sanne E. Hoeks[1*]

[1]Department of Anesthesiology, Erasmus MC University Medical Center, Rotterdam, the Netherlands
[2]Department of Internal Medicine, Division of Geriatric Medicine, Erasmus MC University Medical Center, Rotterdam, the Netherlands
[3]Department of Surgery, Zuyderland Medical Center, Geleen, the Netherlands

© 2019. Tjeertes EKM et al. Published online at http://www.aginganddisease.org/EN/10.14336/AD.2019.1024

SUPPLEMENTARY DATA

Supplementary Table 1. Frailty assessment tools in detail.

Instrument	Description	Items	Range	Suggested Cut-off	Studies
**Frailty Index (FI) / Canadian Study of Health and aging frailty index (CSHA-FI) / Frailty index of accumulated deficits (FI-CD)[23]**	Index of accumulated health deficits in the physical, cognitive, functional and social realms (symptoms, signs, disabilities, laboratory, radiographic) out of a list of 70 deficits	70	0-1	Not frail ≤ 0.25<br>Frail > 0.25	[44, 45]
**Groningen Frailty Index/indicator (GFI) [46]**	(Self-reporting) questionnaire in 4 domains: physical, cognitive, social and psychological	15	0-15	Not frail < 4<br>Frail ≥ 4	[47-52]
**Hopkins Frailty Score / Fried Frailty criteria /(Fried's) Frailty phenotype (FP) [25]**	Defining phenotype of frailty by identifying criteria: unintentional weight loss, exhaustion, low energy expenditure, low grip strength, and slowed waking speed	5	0-5	Not frail: 0 (-1)<br>Pre-frail: (1-) 2<br>Frail ≥ 3	[53-63]
**Modified Frailty Index (mFI) [22]**	Modification of the CSHA-FI, 11 variables: diabetes mellitus, functional status index of ≥ 2, COPD or pneumonia, congestive heart failure, myocardial infarction, percutaneous coronary intervention and/or stenting or angina, hypertension requiring medication, peripheral vascular disease or ischemic rest pain, impaired sensorium, TIA or CVA, and CVA with deficit	11	0-1	Not frail: 0<br>Intermediate frail: 0.09-0.18<br>Frail ≥ 0.27	[33, 35, 36, 64-84]
**(Reported) Edmonton Frail Scale ((R)EFS) [85]**	Questionnaire/test on 9 domains (mood, functional independence, medication use, social support, nutrition, general health status, continence); two performance-based test: Clock test for cognitive impairment and 'Timed Get Up and Go' for functional performance	11	0-17	Not frail: 0-5<br>Vulnerable: 6-7<br>Frail, mildly: 8-9<br>Frail, moderately: 10-11<br>Frail, severely: 12-17	[53, 86, 87]
**Rockwood 7-point Cical Frailty Scale [88]**	7- point clinical frailty score derived from the Canadian Study of Health and Ageing (CSHA)	7	0-7	fit 1; Well 2; Well, treated comorbid disease 3; Apparently vulnerable 4; Mildly frail 5; Moderately frail 6; Severely frail 7	[52, 89-91]
**VES-13; Vulnerable Elderly Survey [92]**	Self-administrated questionnaire concerning perception of health status, example of everyday activity, difficulty performing activities related to health or physical condition	13	0-15	Not frail < 3<br>Frail ≥ 3	[48, 51, 52, 93]
**John Hopkins' Adjusted Clinical Groups (ACG) case-mix system / ACG frailty**	Measure of frailty developed to be used specifically on administrative data Assesses frailty by the presence of 11 conditions (e.g., difficulty walking, weight loss, frequent falls,	12	0-12	Not frail 0<br>Frail ≥ 1	[95-97]

© 2019. Tjeertes EKM et al. Published online at http://www.aginganddisease.org/EN/10.14336/AD.2019.1024

**diagnoses indicator/Johns Hopkins ACG frailty Indicator [94]**	malnutrition, impaired vision, decubitus ulcer, incontinence and 4 unnamed criteria.)				
**Frailty-based bedside risk analysis index (RAI) [98]**	Assessment scoring sex, age, cancer, co-morbidities, residence, cognition, ADL	11	0-75	Not frail < 10<br>Frail ≥ 10	[99]
**Comprehensive geriatric assessment / CGA / Balducci-score [100]**	A multidimensional process that identifies medical, psychosocial, and functional limitations of a frail elderly person	4-6	0-4(/6)	Not frail 0<br>Frail ≥ 1	[52, 101, 102]
**Multidimensional Frailty Score [103]**	Scoring index based on results of the CGA, patient characteristics and laboratory variables	9	0-15	Not frail ≤ 5<br>Frail > 5	[103]

Supplementary information. Full literature search.

Embase.com
('frail elderly'/de OR ((frail* NEAR/6 (elderl* OR centenarian* OR nonagenarian* OR octogenarian* OR septagenarian* OR '65 year*' OR aging OR ageing OR aged OR senior* OR geriatric* OR old OR older))):ab,ti OR ((frail*):ab,ti AND (aged/exp OR aging/de OR 'geriatrics'/de OR 'geriatric care'/de OR 'geriatric assessment'/de))) AND ('abdominal surgery'/exp OR 'breast surgery'/exp OR 'cancer surgery'/exp OR 'ear nose throat surgery'/exp OR 'endocrine surgery'/exp OR 'eye surgery'/exp OR 'head and neck surgery'/exp OR 'major surgery'/exp OR 'neurosurgery'/exp OR 'orthopedic surgery'/exp OR 'plastic surgery'/exp OR 'esophagus surgery'/exp OR 'lung surgery'/exp OR 'transplantation'/de OR 'organ transplantation'/de OR 'kidney transplantation'/de OR 'liver transplantation'/de OR 'lung transplantation'/de OR 'urologic surgery'/exp OR 'vascular surgery'/de OR 'aneurysm surgery'/exp OR 'aorta surgery'/de OR 'artery surgery'/de OR 'carotid artery surgery'/exp OR Endarterectomy/exp OR ('artery surgery'/exp NOT 'coronary artery surgery'/exp) OR 'carotid angioplasty'/exp OR 'carotid artery stenting'/exp OR 'blood vessel shunt'/de OR 'leg revascularization'/exp OR 'embolectomy'/exp OR 'endovascular surgery'/exp OR 'limb salvage'/exp OR 'microvascular surgery'/exp OR 'vein surgery'/exp OR ((('non cardiac' OR noncardiac OR abdominal* OR breast* OR cancer* OR ear* OR nose* OR throat* OR endocrine* OR eye* OR head* OR neck* OR orthopedic* OR plastic* OR urologic* OR esophag* OR oesophag* OR lung* OR pulmonar* OR gastrointestin* OR intestin* OR gastric OR vascul* OR joint* OR renal OR kidney OR hepatic OR liver OR pancrea* OR urologic* OR aneurysm* OR aort* OR carotid* OR colorect* OR orthognat* OR breast* OR neoplasm* OR otorhinolar* OR endocrin* OR ophtalmol* OR orthoped* OR plastic* OR urolog*) NEAR/3 (surg* OR operat* OR perioperat*)) OR neurosurg* OR 'general surgery' OR ((organ* OR kidney* OR liver* OR hepatic* OR renal Or pulmonar*) NEAR/3 transplantat*)):ab,ti)

Medline Ovid
("Frail Elderly"/ OR ((frail* ADJ6 (elderl* OR centenarian* OR nonagenarian* OR octogenarian* OR septagenarian* OR "65 year*" OR aging OR ageing OR aged OR senior* OR geriatric* OR old OR older))).ab,ti. OR ((frail*).ab,ti. AND (exp aged/ OR exp aging/ OR "Geriatrics"/ OR "Geriatric Assessment"/))) AND (Surgical Procedures, Operative/ OR Specialties, Surgical/ OR Colorectal Surgery/ OR General Surgery/ OR Orthognathic Surgery/ OR breast/su OR exp neoplasms/su OR exp Otorhinolaryngologic Diseases/su OR Endocrine Surgical Procedures/ OR exp Ophthalmologic Surgical Procedures/ OR exp neurosurgery/ OR exp Neurosurgical Procedures/ OR Orthopedics/su OR Surgery, Plastic/ OR exp Pulmonary Surgical Procedures/ OR transplantation/ OR organ transplantation/ OR kidney transplantation/ OR liver transplantation/ OR lung transplantation/ OR exp Urologic Surgical Procedures/ OR Vascular Surgical Procedures/ OR Endarterectomy/ OR embolectomy/ OR limb salvage/ OR Microvascular Decompression Surgery/ OR Neurosurgical Procedures/ OR (((non cardiac OR noncardiac OR abdominal* OR breast* OR cancer* OR ear* OR nose* OR throat* OR endocrine* OR eye* OR head* OR neck* OR orthopedic* OR plastic* OR thora* OR urologic* OR esophag* OR oesophag* OR lung* OR pulmonar* OR gastrointestin* OR intestin* OR gastric OR vascul* OR joint* OR renal OR kidney OR hepatic OR liver OR pancrea* OR urologic* OR aneurysm* OR aort* OR carotid* OR colorect* OR general* OR orthognat* OR breast* OR neoplasm* OR otorhinolar* OR endocrin* OR ophtalmol* OR orthoped* OR plastic* OR urolog*) ADJ3 (surg* OR operat* OR perioperat*)) OR neurosurg* OR ((organ* OR kidney* OR liver* OR hepatic* OR renal Or pulmonar*) ADJ3 transplantat*)).ab,ti.)

Cochrane

© 2019. Tjeertes EKM et al. Published online at http://www.aginganddisease.org/EN/10.14336/AD.2019.1024

SUPPLEMENTARY DATA

(((frail* NEAR/6 (elderl* OR centenarian* OR nonagenarian* OR octogenarian* OR septagenarian* OR '65 year*' OR aging OR ageing OR aged OR senior* OR geriatric* OR old OR older))):ab,ti) AND (((('non cardiac' OR noncardiac OR abdominal* OR breast* OR cancer* OR ear* OR nose* OR throat* OR endocrine* OR eye* OR head* OR neck* OR orthopedic* OR plastic* OR urologic* OR esophag* OR oesophag* OR lung* OR pulmonar* OR gastrointestin* OR intestin* OR gastric OR vascul* OR joint* OR renal OR kidney OR hepatic OR liver OR pancrea* OR urologic* OR aneurysm* OR aort* OR carotid* OR colorect* OR orthognat* OR breast* OR neoplasm* OR otorhinolar* OR endocrin* OR ophtalmol* OR orthoped* OR plastic* OR urolog*) NEAR/3 (surg* OR operat* OR perioperat*)) OR neurosurg* OR 'general surgery' OR ((organ* OR kidney* OR liver* OR hepatic* OR renal Or pulmonar*) NEAR/3 transplantat*)):ab,ti)

Web of science
TS=((((frail* NEAR/5 (elderl* OR centenarian* OR nonagenarian* OR octogenarian* OR septagenarian* OR "65 year*" OR aging OR ageing OR aged OR senior* OR geriatric* OR old OR older)))) AND (((("non cardiac" OR noncardiac OR abdominal* OR breast* OR cancer* OR ear* OR nose* OR throat* OR endocrine* OR eye* OR head* OR neck* OR orthopedic* OR plastic* OR urologic* OR esophag* OR oesophag* OR lung* OR pulmonar* OR gastrointestin* OR intestin* OR gastric OR vascul* OR joint* OR renal OR kidney OR hepatic OR liver OR pancrea* OR urologic* OR aneurysm* OR aort* OR carotid* OR colorect* OR orthognat* OR breast* OR neoplasm* OR otorhinolar* OR endocrin* OR ophtalmol* OR orthoped* OR plastic* OR urolog*) NEAR/2 (surg* OR operat* OR perioperat*)) OR neurosurg* OR "general surgery" OR ((organ* OR kidney* OR liver* OR hepatic* OR renal Or pulmonar*) NEAR/2 transplantat*))))

PubMed publisher
("Frail Elderly"[mh] OR ((frail*[tiab] AND (elderl*[tiab] OR centenarian*[tiab] OR nonagenarian*[tiab] OR octogenarian*[tiab] OR septagenarian*[tiab] OR 65 year*[tiab] OR aging OR ageing OR aged OR senior*[tiab] OR geriatric*[tiab] OR old OR older))) OR ((frail*[tiab]) AND (aged[mh] OR aging[mh] OR "Geriatrics"[mh] OR "Geriatric Assessment"[mh]))) AND (Surgical Procedures, Operative[mh] OR Specialties, Surgical[mh] OR Colorectal Surgery[mh] OR General Surgery[mh] OR Orthognathic Surgery[mh] OR breast/su[mh] OR neoplasms/su[mh] OR Otorhinolaryngologic Diseases/su[mh] OR Endocrine Surgical Procedures[mh] OR Ophthalmologic Surgical Procedures[mh] OR neurosurgery[mh] OR Neurosurgical Procedures[mh] OR Orthopedics/su[mh] OR Surgery, Plastic[mh] OR Pulmonary Surgical Procedures[mh] OR transplantation[mh] OR organ transplantation[mh] OR kidney transplantation[mh] OR liver transplantation[mh] OR lung transplantation[mh] OR Urologic Surgical Procedures[mh] OR Vascular Surgical Procedures[mh] OR Endarterectomy[mh] OR embolectomy[mh] OR limb salvage[mh] OR Microvascular Decompression Surgery[mh] OR Neurosurgical Procedures[mh] OR (((non cardiac OR noncardiac OR abdominal*[tiab] OR breast*[tiab] OR cancer*[tiab] OR ear[tiab] OR ears[tiab] OR nose*[tiab] OR throat*[tiab] OR endocrine*[tiab] OR eye*[tiab] OR head*[tiab] OR neck*[tiab] OR orthopedic*[tiab] OR plastic*[tiab] OR thoracic[tiab] OR thorax[tiab] OR urologic*[tiab] OR esophag*[tiab] OR oesophag*[tiab] OR lung*[tiab] OR pulmonar*[tiab] OR gastrointestin*[tiab] OR intestin*[tiab] OR gastric OR vascul*[tiab] OR joint*[tiab] OR renal OR kidney OR hepatic OR liver OR pancreatic*[tiab] OR pancreas*[tiab] OR urologic*[tiab] OR aneurysm*[tiab] OR aort*[tiab] OR carotid*[tiab] OR colorect*[tiab] OR general*[tiab] OR orthognat*[tiab] OR breast*[tiab] OR neoplasm*[tiab] OR otorhinolar*[tiab] OR endocrin*[tiab] OR ophtalmol*[tiab] OR orthoped*[tiab] OR plastic*[tiab] OR urolog*[tiab]) AND (surg*[tiab] OR operation[tiab] OR operative[tiab] OR perioperat*[tiab])) OR neurosurg*[tiab] OR ((organ[tiab] OR organs[tiab] OR kidney*[tiab] OR liver*[tiab] OR hepatic*[tiab] OR renal Or pulmonar*[tiab]) AND transplantat*[tiab]))) AND publisher[sb]

Google scholar
200 first results: Frail|frailty elderly|older|seniors "non cardiac surgery"|"noncardiac surgery"
All results: allintitle:Frail|frailty elderly|older|seniors surgery|operative -cardiac

Supplementary Table 2. Quality assessment of included studies.

Study	Study participation	Study attrition	Prognostic factor measurement	Outcome measurement	Confounding measurement and account	Analysis	Overall rating
**Abt**	+	+	+	+	±	+	11
**Adams**	+	+	+	+	±	+	11
**Arya**	+	+	+	+	+	+	12
**Augustin**	+	+	+	+	+	+	12
**Brahmbhatt**	+	+	+	+	+	+	12
**Bras**	±	±	+	+	**?**	±	8

© 2019. Tjeertes EKM et al. Published online at http://www.aginganddisease.org/EN/10.14336/AD.2019.1024

SUPPLEMENTARY DATA

**Chappidi**	+	+	+	+	+	+	12
**Chimukangara**	±	+	+	+	±	±	9
**Cloney**	+	±	+	+	-	±	8
**Cooper**	+	+	+	±	-	±	8
**Courtney-Brooks**	±	+	+	+	-	±	8
**Dale**	±	+	±	+	±	±	8
**Dasgupta**	±	±	+	+	+	+	10
**Farhat**	+	+	±	+	?	+	9
**Flexman**	+	+	+	+	+	±	11
**Hewitt**	±	+	+	+	±	±	9
**Huisman**	+	±	+	+	+	+	11
**Joseph**	±	±	+	+	±	+	9
**Kenig**	±	±	+	±	+	+	9
**Kim** (2016)	+	+	+	±	+	+	11
**Kim** (2014)	+	±	+	+	+	+	11
**Krishnan**	±	±	±	±	-	±	6
**Kristjansson**	+	+	+	+	+	±	11
**Kua**	±	?	+	+	±	+	8
**Lascano**	+	+	+	+	+	+	12
**Lasithiotakis**	±	?	±	+	-	±	5
**Leung**	-	±	+	±	+	+	8
**Levy**	+	+	+	+	+	+	12
**Li**	+	±	±	+	+	±	9
**Louwers**	+	+	+	+	±	+	11
**Makary**	+	+	+	+	±	+	11
**McAdams-DeMarco**	±	+	+	+	+	±	10
**McIsaac (JAMA)**	+	+	+	+	+	+	12
**McIsaac**	+	+	+	+	+	+	12
**Melin**	+	+	+	+	-	-	8
**Mogal**	+	+	+	+	+	+	12
**Mosquera**	+	+	±	+	?	+	9
**Neuman**	+	±	±	+	±	+	9
**Obeid**	+	+	+	+	±	±	10
**Partridge**	±	±	+	+	±	+	9
**Pearl**	+	+	+	+	±	+	11
**Phan**	+	+	+	+	+	+	12
**Reisinger**	+	+	+	+	±	+	11
**Revenig (2015)**	+	+	+	+	±	±	10
**Revenig (2014)**	±	+	+	+	±	+	10
**Revenig (2013)**	±	+	+	+	+	+	11

© 2019. Tjeertes EKM et al. Published online at http://www.aginganddisease.org/EN/10.14336/AD.2019.1024

SUPPLEMENTARY DATA

**Robinson**	+	+	±	+	-	±	8
**Shin (2017)**	+	+	+	+	+	+	12
**Shin (2016)**	+	+	+	+	+	+	12
**Suskind**	+	+	+	+	+	+	12
**Suskind (Urology)**	+	+	+	+	+	+	12
**Tan**	±	+	+	+	±	+	10
**Tegels**	±	±	+	+	±	+	9
**Tsiouris**	+	+	+	+	±	+	11
**Ugolini**	±	±	+	+	±	-	7
**Uppal**	+	+	+	+	+	+	12

Study participation: The study correctly defines and describes the study population
Study attrition: The study was able to obtain a complete follow up
Prognostic factor management: The study provides a clear description of the prognostic factor measured
Outcome measurement: The study provides a clear definition of outcome
Confounding measurement and account: Adequately valid and reliable measurement of potential confounders
Analysis: The statistical analysis is appropriate for the design of the study

Description of used symbols:
+ Yes; adequate and complete description
± Partly; incomplete description
? Unsure; doubtful description
- No; not described

© 2019. Tjeertes EKM et al. Published online at http://www.aginganddisease.org/EN/10.14336/AD.2019.1024
